# Supplementary material for: Interplay between Systemic Glycemia and Neuroprotective Activity of Resveratrol in Modulating Astrocyte SIRT1 Response to Neuroinflammation
Source: Int J Mol Sci. 2023 Jul 19;24(14):11640. doi: 10.3390/ijms241411640 (PMC10380505; doi:10.3390/ijms241411640)
Supplement: Supplementary file 1 [file ijms-24-11640-s001.zip › ijms-2448051-supplementary.pdf]

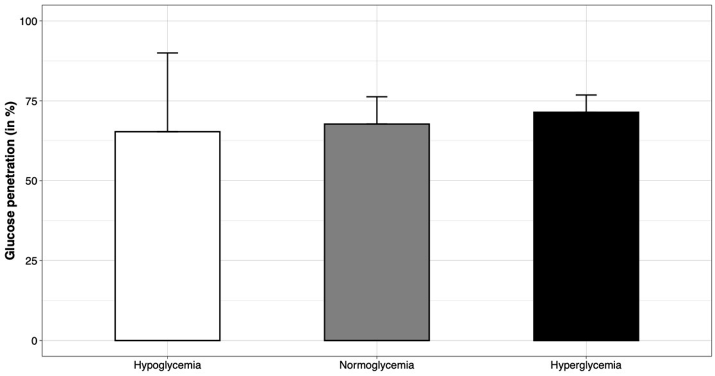

Supplementary Figure S1. Glucose penetration through the blood-brain barrier model used in the present study assessed by colorimetric assay in three different glycemic backgrounds (hypo-, normo- and hyperglycemia, resulting from applying 2.2 mM, 5 mM and 25 mM glucose to the microvascular compartment, respectively).

Analogically to the two-cell blood-brain barrier (BBB) model used in this study, consisting of the astrocyte brain compartment (BC) and the endothelial microvascular compartment (MC), the endothelial MC and astrocyte-free glucose-free BC were maintained for 24 hours and subsequently for 24 hours following glucose addition to the MC in concentrations corresponding to hypo-, normo- and hyperglycemia. At this time (corresponding to the experimental time point 0h) glucose concentration in the BC was measured using colorimetric assay (for details see section *4.5*) and the ratio of glucose penetration through the BBB was calculated. Mean values of glucose penetration ratio (in %) and standard errors are presented. There were no statistically significant differences in the ratio of glucose penetration through the BBB between different glycemic groups.

Supplementary Table S1. Statistical measures of central tendency and variability for the sirtuin 1 concentration data. Mean values (Mean) and standard deviations (SD) for all the data on sirtuin 1 (SIRT1) concentration, presented in Figures 2, 4-9. As the data for time point 0h were pooled across the experiments, at this time point n=12 for each glycemic group. The data for time point 12h were pooled for untreated with RSV-treated and for LPS-treated with LPS-RSV-treated groups, thus at this time point n=6 for each glycemic group. For all other time points and conditions n=3.

Glycemic background	Time point	untreated (∅)	+LPS	+RSV	+LPS +RSV
Hypoglycemia	0h	Mean: 211.000<br>SD: 120.032	Mean: 211.000<br>SD: 120.032	Mean: 211.000<br>SD: 120.032	Mean: 211.000<br>SD: 120.032
	12h	Mean: 185.000<br>SD: 125.709	Mean: 200.167<br>SD: 237.981	Mean: 185.000<br>SD: 125.709	Mean: 200.167<br>SD: 237.981
	24h	Mean: 317.000<br>SD: 89.163	Mean: 173.500<br>SD: 10.607	Mean: 254.000<br>SD: 199.404	Mean: 191.000<br>SD: 188.090
	36h	Mean: 364.000<br>SD: 57.000	Mean: 13.000<br>SD: 8.000	Mean: 326.500<br>SD: 160.450	Mean: 86.500<br>SD: 86.974
Normoglycemia	0h	Mean: 197.375<br>SD: 123.628	Mean: 197.375<br>SD: 123.628	Mean: 197.375<br>SD: 123.628	Mean: 197.375<br>SD: 123.628
	12h	Mean: 214.667<br>SD: 144.119	Mean: 224.250<br>SD: 41.153	Mean: 214.667<br>SD: 144.119	Mean: 224.250<br>SD: 41.153
	24h	Mean: 190.667<br>SD: 68.857	Mean: 152.333<br>SD: 124.001	Mean: 240.333<br>SD: 154.730	Mean: 189.000<br>SD: 55.154
	36h	Mean: 181.000<br>SD: 53.703	Mean: 43.000<br>SD: 21.213	Mean: 266.250<br>SD: 148.744	Mean: 144.500<br>SD: 94.045
Hyperglycemia	0h	Mean: 206.125<br>SD:150.088	Mean: 206.125<br>SD:150.088	Mean: 206.125<br>SD:150.088	Mean: 206.125<br>SD:150.088
	12h	Mean: 144.250<br>SD: 75.769	Mean: 195.000<br>SD: 66.106	Mean: 144.250<br>SD: 75.769	Mean: 195.000<br>SD: 66.106
	24h	Mean: 132.500<br>SD: 88.138	Mean: 13.000<br>SD: 8.000	Mean: 208.500<br>SD: 38.891	Mean: 32.000<br>SD: 15.556
	36h	Mean: 57.667<br>SD: 25.502	Mean: 43.000<br>SD: 18.000	Mean: 166.000<br>SD: 12.728	Mean: 113.500<br>SD: 48.790
